# Transcriptomic Analysis of the Primary Roots of *Alhagi sparsifolia* in Response to Water Stress

**DOI:** 10.1371/journal.pone.0120791

**Published:** 2015-03-30

**Authors:** Huanian Wu, Yongqiang Zhang, Wangbin Zhang, Xinwu Pei, Chao Zhang, Shirong Jia, Weimin Li

**Affiliations:** 1 Biotechnology Research Institute, Chinese Academy of Agricultural Sciences, 12 Zhongguancun South Street, Beijing 100081, PR China; 2 College of plant science, Tarim University, Alar, Xinjiang 843300, PR China; Estación Experimental del Zaidín (CSIC), SPAIN

## Abstract

**Background:**

*Alhagi sparsifolia* is a typical desert phreatophyte and has evolved to withstand extreme dry, cold and hot weather. While *A*. *sparsifolia* represents an ideal model to study the molecular mechanism of plant adaption to abiotic stress, no research has been done in this aspect to date. Here we took advantage of Illumina platform to survey transcriptome in primary roots of *A*. *sparsifolia* under water stress conditions in aim to facilitate the exploration of its genetic basis for drought tolerance.

**Methodology and Principal Findings:**

We sequenced four primary roots samples individually collected at 0, 6, 24 and 30h from the *A*. *sparsifolia* seedlings in the course of 24h of water stress following 6h of rehydration. The resulting 38,763,230, 67,511,150, 49,259,804 and 54,744,906 clean reads were pooled and assembled into 33,255 unigenes with an average length of 1,057 bp. All-unigenes were subjected to functional annotation by searching against the public databases. Based on the established transcriptome database, we further evaluated the gene expression profiles in the four different primary roots samples, and identified numbers of differently expressed genes (DEGs) reflecting the early response to water stress (6h *vs*. 0h), the late response to water stress (24h *vs*. 0h) and the response to post water stress rehydration (30h *vs*. 24h). Moreover, the DEGs specifically regulated at 6, 24 and 30h were captured in order to depict the dynamic changes of gene expression during water stress and subsequent rehydration. Functional categorization of the DEGs indicated the activation of oxidoreductase system, and particularly emphasized the significance of the ‘Glutathione metabolism pathway’ in response to water stress.

**Conclusions:**

This is the first description of the genetic makeup of *A*. *sparsifolia*, thus providing a substantial contribution to the sequence resources for this species. The identified DEGs offer a deep insight into the molecular mechanism of *A*. *sparsifolia* in response to water stress, and merit further investigation.

## Introduction

Drought stress due to water scarcity has emerged as one of the most urgent issues facing terrestrial ecosystem in many areas of the world to date. Under water deficit conditions, plants often show an array of morphological, physiological and biochemical changes, which may negatively impact plant germination, growth, development as well as reproduction, leading to substantial losses in both natural ecosystem and agricultural production [1,2]. Thus, it is essential to develop plants with improved drought tolerance as well as water use efficiency through various genetic approaches. To this end, a comprehensive understanding of genetic basis of drought tolerance in plants is significant and imperative [3,4].

Plants, due to their sessile nature, have to evolve various mechanisms to cope with water deficit stress, thereby enabling themselves to adapt and survive in the course of drought. Thus far, many progresses have been made toward unraveling the molecular networks that plants use to defense against water stress, and a large array of transcription factors (TFs) as well as signaling mediators including hormones, ion, reactive oxygen species (ROS), protein kinases and phosphatases have been identified to together form delicate signaling cascades in response to water stress [5–8]. For instance, the stomatal closing, one of the well-studied responses in plants to drought stress, is known to be mediated with a well-orchestrated integration of ABA, nitric oxide, ROS (particularly H_2_O_2_), Ca^2+^, phosphatases [5, 9–11]. Recently, it was found that many different types of TFs such as NAC, MYB and WRKY were also involved in this biological process [9,12,13].

Plant roots are important organs growing underground. In addition to absorption and transport of soil water and nutrients for maintaining plant growth, roots are known to serve as one of the primary sites to perceive and monitor soil water content [14,15]. Once the edaphic water deficit occurs, an array of root-sourced chemical and hydraulic signals are triggered and transported to shoot, allowing timely initiation of an integrated cascade of gene expression responses to water stress [16–18]. Regarding the significance of root sensing and response of water deficit, it has become one important aspect to underly how plants adapt to drought stress [19]. Over the past decades, considerable research and interest have been focused on the gene expression profiles in roots of a broad range of plants under drought stress, such as Arabidopsis [20], *Ammopiptanthus mongolicus* [21], chickpea [22], common bean [23], cotton [24,25], sunflower [26], maize [27], pine [28], poplar [29] and rice [30]. However, the mechanisms for water stress signaling and adaptation in roots remain elusive, additional characterization of signal transduction and gene networks controlling the response of roots to water stress is still necessary.

*Alhagi sparsifolia* Shap. is a perennial subshrub belonging to genus *Alhagi*, family *Leguminosae*. As a typical desert phreatophyte, this plant species has highly developed deep roots and displays a great capacity to withstand poor soil as well as extreme dry, cold and hot weather [31]. *A*. *sparsifolia* is naturally distributed in the arid and salinized regions of northernwestern China and adjacent countries in Central Asia, and plays a fundamental role in maintaining the local ecosystem [32]. In addition, this plant is usually used as a fodder for local animals due to its high protein content, and is important in the development of local livestock husbandry [33]. Considering its great tolerance to harsh environments, *A*. *sparsifolia* represents an ideal species for deciphering the mechanism of plant adaption to abiotic stress, such as water deficit. However, understanding the stress adaption in this plant is limited. While a few studies were conducted on physiological responses of *A*. *sparsifolia* to water stress [31,34,35], research has never been done to explore the genomic basis for its drought-tolerance to date. In particular, as a non-model plant species, the genomic resource of *A*. *sparsifolia* is rather scarce, only one expressed sequence tag (EST) and one protein have been deposited in Genbank prior to Sept., 2014.

The development of primary roots of seedlings is a principal component that has been strongly associated with drought tolerance in terrestrial plants. In this study, to gain a deep insight into the drought tolerance in *A*. *sparsifolia*, we then focused on its primary roots and adopted RNA sequencing (RNA-seq), which provide a unique opportunity for genomic exploration in non-mode species [36], to survey the gene expression profiles of primary roots under water stress conditions. We sequenced four cDNA libraries which represent different primary root tissues sampled at 0h, 6h, 24h and 30h during 24h of water stress following 6h of rehydration, and obtained a total of 21.04 gigabase pairs of clean reads. After *de novo* assembly and gene annotation, a primary root transcriptome database of *A*. *sparsifolia* containing 33,255 unigenes was established. Furthermore, on the basis of the transcriptome analysis, the differently expressed genes (DEGs) reflecting the early or late responses to water stress, and the response to post stress rehydration in *A*. *sparsifolia* primary roots were identified. To our knowledge, this is the first transcriptome database of *A*. *sparsifolia*, which not just shed light on the mechanisms of its adaptation to drought stress, but lay the foundation for future functional genomics studies on this species.

## Materials and Methods

### Plant materials and measurement of lengths of the primary roots under water stress

The *A*. *sparsifolia* seeds were collected from Taklamakan, North-west China with the permit from Forestry Department of Xinjiang Uygur Autonomous Regions. To facilitate their germination, the seeds with similar size were first treated with concentrated sulfuric acid (98%) for 20 min, and immersed in sterile distilled water for 30 min preceded by 6 rinses with distilled water. After that, the seeds were placed in 12 cm Petri dishes with 2-layer fully wetted filter paper, and cultured in the dark at 25°C for 24 h. The properly germinated seeds were then selected and transferred to Petri dishes containing 2-layer filter paper saturated with the different percentages (90, 150, 220, 270 and 320 g/L) of polyethylene glycol-6000 (PEG-6000) solution, respectively. Notably, the osmotic potential (ψs) value of the series of PEG solutions was of -0.13, -0.30, -0.58, -0.85 and -1.24MPa, as calculated according to Michel and Kaufmalln [37]. The sterile distilled water was used as a control. The seedlings were remained at 25°C in the dark for 3 days followed by 4 additional days under 16-h light/8-h dark. During the 7-day culture period, water losses due to evaporation were calculated by weighing Petri dishes containing paper and seedlings for every 12 h, and sterile distilled water was added as necessary to compensate for the evaporation losses. At least 15 germinated seeds were included in each different treatment, and three independent experiments were preformed. The typical *A*. *sparsifolia* seedlings were photographed 7 days post treatment with PEG-6000 and lengths of their primary roots were measured.

### Preparation of the primary root samples

The *A*. *sparsifolia* seeds were first treated with concentrated sulfuric acid (98%) and placed in Petri dishes for germination as described above. After 2 days, the well developed *A*. *sparsifolia* seedlings were selected and placed into 12 cm Petri dishes with 2-layer filter paper saturated with the 22% PEG-6000 solution in the dark. At 0, 6 and 24 h post treatment, the primary roots were collected to generate an untreated sample (P-0h) as well as two other samples (P-6h and P-24h) that individually represent the early and late responses to water stress in primary roots. Moreover, the seedlings with 24 h of PEG treatment were thoroughly rinsed with sterile distilled water, and then re-transferred to Petri dishes but containing moist filter paper, and cultured for additional 6 h in the dark (in total, up to 30h of continuous treatment) to make the sample Rh-6h representing the response to post stress rehydration in primary roots. About 40 seedlings were used in each different treatment, and three independent experiments were performed. The samples were immediately frozen in liquid nitrogen and were stored at -80°C until use.

### cDNA library preparation for transcriptome sequencing

Total RNA was extracted from P-0h, P-6h, P-24h and Rh-6h, respectively, by using TRIzol reagent (Invitrogen, USA). To prepare cDNA samples for transcriptome sequencing, total RNA was first assessed with NanoPhotometer spectrophotometer (IMPLEN, USA) and RNA Nano 6000 Assay Kit of the Agilent Bioanalyzer 2100 system (Agilent Technologies, USA) to ensure the quality and integrity, and then processed with NEBNext Ultra RNA Library Prep Kit for Illumina (NEB, USA) following manufacturer’s instruction. Briefly, poly(A) mRNA was first isolated from 3 μg of total RNA using oligo (dT) magnetic beads (Invitrogen, USA), and then was fragmented into smaller pieces at 70°C for 5min in the fragmentation buffer (Ambion, USA). Taking these mRNA fragments as templates, the first-strand cDNA was synthesized with M-MuLV Reverse Transcriptase (RNase H^-^) and random hexamer primer, and second strand cDNA synthesis was subsequently performed using DNA polymerase I (Invitrogen, USA) and RNaseH (Invitrogen, USA). The resulting double-stranded cDNA fragments were purified with AMPure XP system (Beckman Coulter, USA) followed by end repair, 3′ poly(A) addition, and NEBNext Adaptor ligation. The cDNA fragments of ~200 bp in length were selected and further enriched with PCR amplification to generate the cDNA libraries of P-0h, P-6h, P-24h and Rh-6h for Illumina sequencing. The library quality was evaluated on the Agilent Bioanalyzer 2100 system.

### Clustering and sequencing

The clustering of the index-coded samples was performed on a cBot Cluster Generation System with TruSeq PE Cluster Kit v3-cBot-HS (Illumia, San Diego, USA) following the manufacturer’s instructions. After that, the cDNA library was loaded onto the channels of an Illumina HiSeq 2000 for in-depth sequencing. The fluorescent images deconvolution and quality value calculation were performed using the Illumina data processing pipeline (version 1.6), in which 100 bp paired-end reads were generated.

### Data filtering and *de novo* assembly by Trinity

Before assembly, the raw reads were first processed through in-house perl scripts to filter high-quality clean reads by removing low quality reads as well as reads containing adaptor or unknown nucleotides larger than 10%. Clean reads derived from libraries of P-0h, P-6h, P-24h and Rh-6h were pooled and *de novo* assembled into transcripts using Trinity method [38] with optimized K-mer length of 25 and coverage cut-off value of 2. For removing redundancy, when a component contained more than one assembled transcript, only the longest one was preserved to represent an unigene.

To determine the sequence direction, the generated unigenes were aligned by blastx (E-value≤1e-5) to protein databases with the priority order of NCBI NR (non-redundant protein sequence), Swiss-Prot (A manually annotated and reviewed protein sequence database), KEGG (Kyoto Encyclopedia of Genes and Genomes database) and KOG (euKaryotic Ortholog Groups) if conflicting results were obtained. ESTScan 3.03 [39] was also used to determine the direction of the sequence when a unigene was not aligned to any of the databases described above.

### Unigene annotation and classification

For unigene annotation, the assembled unigenes longer than 200 bp were blast against the protein databases of NR, Swiss-Prot, KEGG, KOG as well as the NCBI nucleotide sequences database of NT (non-redundant nucleotide) by using NCBI blastX with a cut-off E-value of ≤1e-5. In addition, the unigenes were also annotated with the protein databases of Pfam (Protein family database) and KEGG by using HMMER 3.0 and KAAS_sa2, respectively.

With NR and Pfam annotation, Blast2GO [40] was employed to obtain GO (Gene Ontology) annotation defined by molecular function, cellular component and biological process ontology. The KOG and KEGG pathways annotation was determined by using Blastall (E-value≤10^-5^) to search against the KOG database and the KEGG pathway database, respectively.

### Identification of unigene expression level and differentially expressed genes

The clean reads from each RNA-seq library were mapped backed onto the assembled transcriptome using RSEM with a maximum of no more than 2 nucleotides mismatches [41]. The resulting readcount for each matched unigene was normalized with FPKM (expected number of Fragments Per Kilobase of transcript sequence per Millions base pairs sequenced) as described [42] to measure its expression level.

Prior to differential gene expression analysis, for each sequenced library, the readcounts were adjusted with edgeR program package [43] through one-scaling normalized factor. Calculation of the differentially expressed genes between two assigned libraries (P-6h *vs*. P-0h, P-24h *vs*. P-0h, Rh-6h *vs*. P-0h and Rh-6h *vs*. P-24h) was performed by using the DEGseq R package [44]. The *p*-value was adjusted using *q*-value [45], and threshold as “*q*-value<0.005 & |log2 (foldchange) |>1” was set to judge the significance of gene expression difference.

The filtered differentially expressed genes (DEGs) were mapped to GO and KEGG database using GOseq [46] and KOBAS [47] to search the significantly enriched GO terms and KEGG pathways, respectively.

### Reverse transcription quantitative real-time PCR (RT-qPCR) analysis

Ten unigenes with different expression patterns were selected for RT-qPCR analysis. In brief, 1.0 μg of total RNA prepared from P-0h, P-6h, P-24h or Rh-6h was first reverse transcribed into its cDNA with oligo(dT)_18_ through M-MLV Reverse Transcriptase (Progema, USA). The qPCR was then performed on an ABI 7500 Real-Time System (Applied Biosystems, USA) with a 25-μL reaction system, which contain 12.5 μL 2× SYBR *Premix EX* Taq (Takara, Japan), 10 pmol forward and reverse primers and 0.5 μL template cDNA. The primer sequences are listed in Table A in S1 File. The PCR conditions were as follows: 50°C for 2 min and 95°C for 10 min; 40 cycles of 95°C for 15 sec and 60°C for 1 min; 1 cycle of 95°C for 15 sec, 60°C for 15 sec and 95°C for 15 sec. *Actin* gene of *A*. *sparsifolia* was monitored as an internal control to normalize template amounts. Three independent replicates were performed for each sample. The relative expression levels of the unigenes were calculated by the relative 2^–ΔΔCT^ method [48]. Results represent mean standard deviations of the three experimental replicates.

### Data deposition

The nucleotide sequences of raw reads as well as the assembled unigenes from this study were submitted to NCBI Gene Expression Omnibus under the accession number GSE62174.

## Results

### Effect of water stress induced by PEG-6000 on elongation of the *A*. *sparsifolia* primary roots

**Fig 1:**
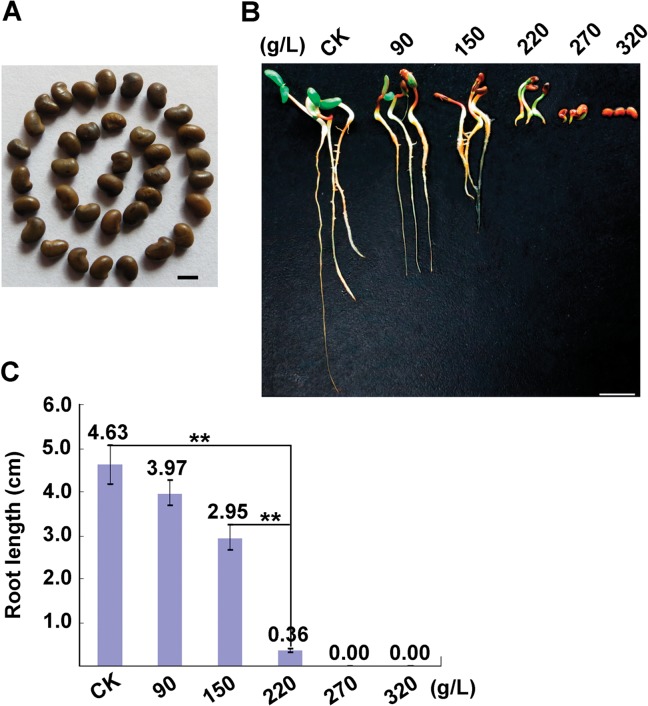
Effect of water stress induced by PEG-6000 on development of the *Alhagi sparsifolia* primary roots. (A) Seeds of *A*. *sparsifolia* (Scale bar = 2.0 cm). (B) Typical phenotype of the *A*. *sparsifolia* seedlings at 7 days post treatment with PEG-6000 (Scale bar = 1.0 cm). The *A*. *sparsifolia* seeds were first treated with concentrated sulfuric acid (98%) for 20 min, and then put on fully wetted filter paper at 25°C in the dark for 24 h. The germinated seeds were selected and transferred to petri dishes containing filter paper saturated with the different percentages of PEG-6000 solution (0, 90, 150, 220, 270 and 320 g/L), and were remained at 25°C in the dark for 3 days followed by 4 additional days but under 16-h light/8-h dark. At least 15 seedlings were included in each different treatment, and three independent experiments were preformed. (C) Average length of the primary roots of *A*. *sparsifolia* treated with PEG-6000 at 7 days. Error bars represent SE. P values determined by Student t test (** p < 0.01).

To examine elongation of the *A*. *sparsifolia* primary root under water stress, the PEG-6000 solution, which enabled us control and repeat the water stress conditions easily and precisely in laboratory settings, was used in this study. The selected *A*. *sparsifolia* seeds (Fig. 1A) were first germinated on the moist filter paper for 24 h, and then exposed to various concentrations of PEG-6000 solutions in the covered Petri dishes for additional 7 days. It was observed that, with increasing concentrations of PEG-6000 from 90 to 320 g/L, elongation of the *A*. *sparsifolia* primary roots became gradually retarded. In particular, the shortest elongation of the primary roots developed at the 220 g/L PEG-6000 solution (Fig. 1B and 1C). Accordingly, the PEG-6000 solution at concentration of 220 g/L was used hereblow to mimic water stress in the *A*. *sparsifolia* seedlings.

### Transcriptome sequencing of the *A*. *sparsifolia* primary roots and *de novo* assembly

The main goal of this study was to gain a global view of the transcriptome response to water stress in primary roots of *A*. *sparsifolia*. To this end, the 2-day-old *A*. *sparsifolia* seedlings, which were first germinated on the water-saturated filter paper, were individually subjected to 0h, 6h, 24h of PEG treatment, and 24h of PEG treatment following 6h of rehydration. Only the primary roots were collected, resulting in four different samples namely P-0h, P-6h, P-24h and Rh-6h that represent the untreated primary roots, the early and late responses of primary roots to water stress, and the response of primary roots to post stress rehydration, respectively. It is notable that the primary roots of the 2-day-old seedling used here were of ~5–8 mm in length, and their elongation almost stagnated under the treatment of 22% PEG-6000, thus making the primary roots of P-0h, P-6h, P-24h and Rh-6h with nearly uniform length. The Illumina sequencing platform HiSeq 2000 was then applied to explore the transcriptome in each of the primary roots samples. As shown in Table 1, there were 52,252,810 raw reads generated from P-0h, 82,109,642 from P-6h, 54,259,176 from P-24h, and 59,886,362 from Rh-6h (GEO accession numbers: GSE62174). These raw reads were further filtered by removing adaptors, ambiguous nucleotides and low-quality sequences to generate clean reads. In total, 38,763,230 clean reads, each of which has the length of 100 bp, remained in the library P-0h, 67,511,150 in P-6h, 49,259,804 in P-24h, and 54,774,906 in Rh-6h (Table 1).

**Table 1:** Overview of the sequencing reads.

Sample^a^	Raw reads	Clean reads^b^	Clean bases	Error (%)	Q20^c^ (%)	Q30^d^ (%)	GC (%)
P0_Root_L1	26,126,405	19,381,615	1.94G	0.03	98.06	93.08	50.27
P0_Root_R2	26,126,405	19,381,615	1.94G	0.05	94.96	88.10	50.23
P6_Root_L1	41,054,821	33,755,575	3.38G	0.03	98.16	93.40	47.47
P6_Root_R2	41,054,821	33,755,575	3.38G	0.04	95.32	88.69	47.39
P24_Root_L1	27,129,588	24,629,902	2.46G	0.03	98.21	93.80	46.79
P24_Root_R2	27,129,588	24,629,902	2.46G	0.06	94.30	87.63	46.79
Rh6_Root_L1	29,943,181	27,387,453	2.74G	0.03	98.25	93.92	46.60
Rh6_Root_R2	29,943,181	27,387,453	2.74G	0.06	94.22	87.43	46.59
Total	248,507,990	210,309,090	21.04G	—	—	—	—

a, L1: Reads sequencing from the left; R2: Reads sequencing from the right.

b, adaptors and low-quality reads were excluded.

c, Q20: The percentage of bases with quality value larger than 20.

d, Q30: The percentage of bases with quality value larger than 30.

**Fig 2:**
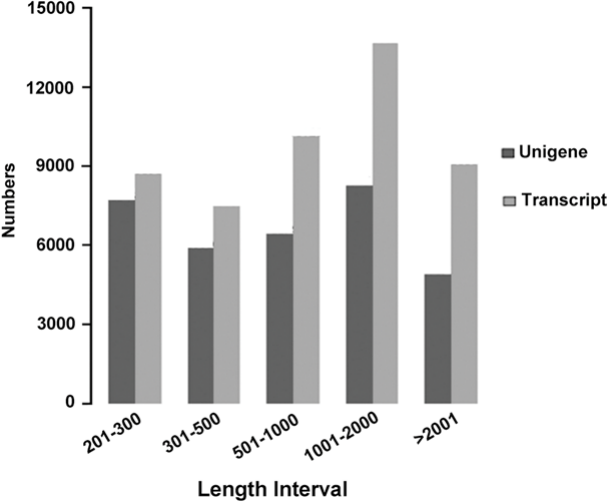
Length frequency distribution of transcripts and unigenes assembled by Trinity. X-axis represents the size of sequences (nt), and Y-axis indicates the number of sequences.

The clean reads yielded from the four different transcriptome libraries were pooled and further processed with Trinity software [38], which was often used to assemble full-length transcripts without reference genomes. A total of 49,051 transcripts ranging from 201 to 15,635 bp were produced. After removal of the redundancy from the assembled transcripts, 33,255 unigenes were achieved (GEO accession numbers: GSE62174) with a total length of 35,143,468 bp and an average length of 1,057 bp (Table 2). The size distribution of the transcripts and unigenes was shown in Fig. 2. The all-unigenes provided a sequence basis for analysis of gene expression in *A*. *sparsifolia* primary roots.

**Table 2:** General features of the primary root transcriptome of *A*. *sparsifolia*.

	Transcript	Unigene
Total number	49,051	33,255
Total nucleotides	59,141,281	35,143,468
Length range	201~15,635	201~15,635
Mean length	1,206	1,057
Median length	903	696
N50^a^	1,866	1,732
N90^b^	568	436

a, N50: the length *L* where 50% of all nucleotides in the assembly are contained in transcripts/unigenes of size≥ *L*.

b, N90: the length *L* where 90% of all nucleotides in the assembly are contained in transcripts/unigenes of size≥ *L*.

To verify the quality of sequencing data, 20 unigenes were selected and pairs of primers were designed accordingly for RT-PCR amplification. Each primer pair produced a PCR product with the expected size and its identity was confirmed by Sanger sequencing (data not shown).

### Unigene annotation to public databases

To predict the putative functions, all of the 33,255 unigenes were searched against the public databases of NR, NT, Swiss-Prot, Pfamm, KOG and KEGG for annotation. Totally 24,639 unigenes (74.09% of all distinct sequences) showed significant matches to NR database, 22,494 (67.64%) unigenes were annotated in NT database and 17,806 (53.54%) in Swiss-Prot database. In addition, 16,417 (49.36%) unigenes encoded similar protein domains or motifs to those in Pfam database (Table 3), and the numbers of unigenes with significant similarity to sequences in KOG and KEGG were of 9,313 (28.0%) and 6,452 (19.4%), respectively. Among all these unigenes, 3,821 (11.49%) were simultaneously annotated by all six databases, and 26,206 (78.80%) showed homology to the known sequences deposited in at least one database (Table 3).

**Table 3:** Summary of unigene annotations against public databases.

	Number of Unigenes	Percentage (%)
Total Unigenes	33,255	100
Gene annotation against NR	24,639	74.09
Gene annotation against NT	22,494	67.64
Gene annotation against SwissPort	17,806	53.54
Gene annotation against Pfam	16,417	49.36
Gene annotation against KOG	9,313	28.0
Gene annotation against KEGG	6,452	19.4
Gene annotation against GO	18,272	54.94
Unigenes annotated in all databases	3,821	11.49
Unigenes annotated in at least one databases	26,206	78.80

Abbreviations: NR, non-redundant protein sequence; NT, non-redundant nucleotide; Pfam, Protein Family Database; KEGG, Kyoto Encyclopedia of Genes and Genomes database; KOG, euKaryotic Ortholog Groups; GO, Gene Ontology.

**Fig 3:**
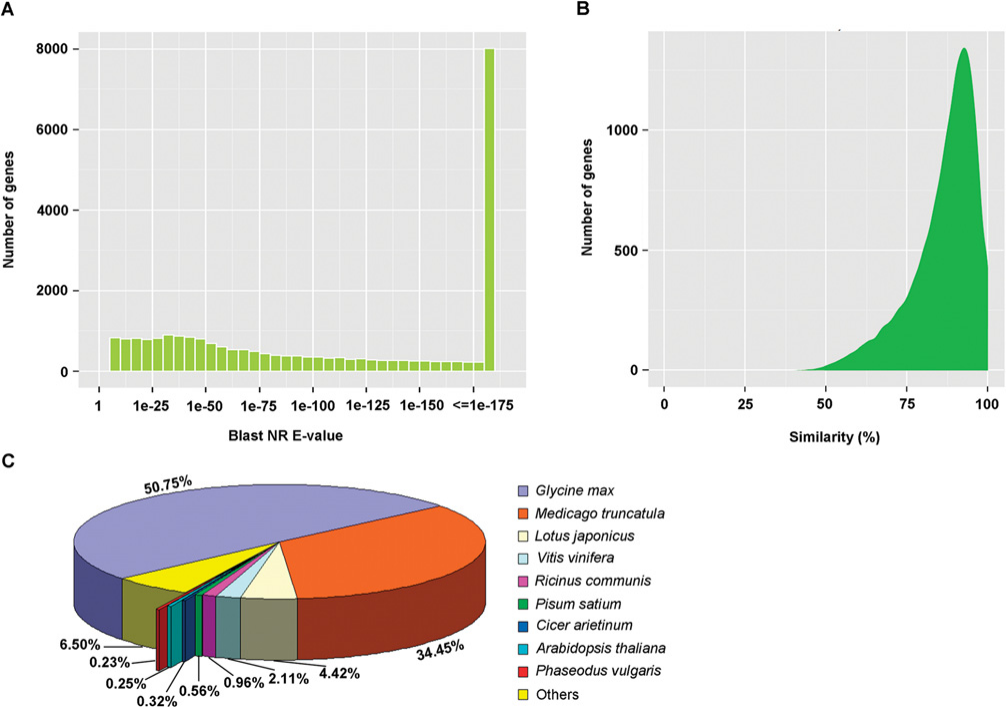
Characteristics of homology search of unigenes against the NR database. (A) E-value distribution of the top BLAST hits for each unigene with a cut-off E-value of 1.0E-5. (B) Similarity distribution of the best BLAST hits for each unigene. (C) Species distribution is shown as the percentage of the total homologous sequences with an E-value of at least 1.0E-5. We used all plant proteins in the NCBI NR database for homology search and extracted the first hit of each sequence for analysis.

The E-value distribution of the top hits in the NR database revealed that 69.4% of the annotated unigenes had strong homology (<1.0E^-50^), and the rest had homology ranging between 1.0E^-5^ to 1.0E^-50^ (Fig. 3A). The similarity distribution showed that 79.6% of the query sequences have a similarity up to 80%, and only a small number (8.7%) of the hits have a similarity ranging from 41% to 70% (Fig. 3B). In consistence with the phylogenetic status of *A*. *sparsifolia* [35], a significant number (>90%) of the annotated unigenes have top matches (first hit) with genes from species in *Leguminosae* family (Fig. 3C), including *Glycine max* (50.2%), *Medicago truncatula* (34.4%) and *Lotus japonicus* (4.4%).

### Functional annotation and classification of unigenes

**Fig 4:**
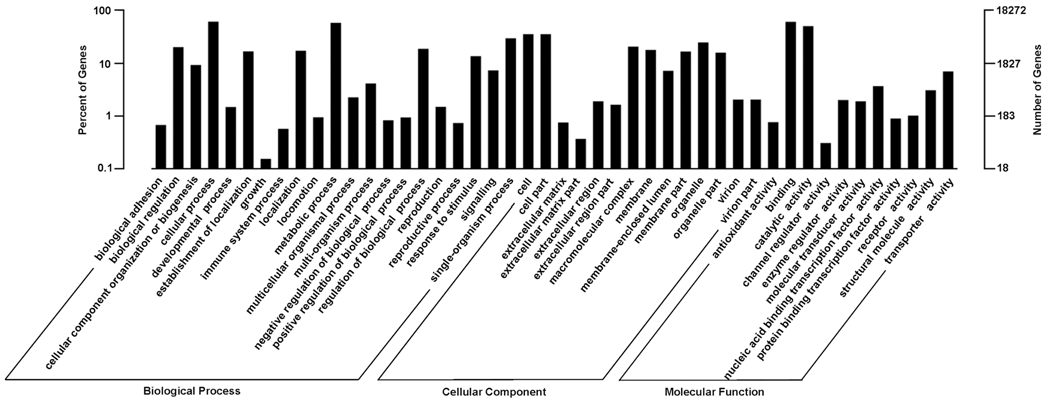
GO categories of biological process, cellular component and molecular function for the transcriptome of the *A*. *sparsifolia* primary roots. The right y-axis shows the number of genes in a category, and the left y-axis indicates the percentage of a specific category of genes in that main category.

GO assignment was used to classify the functions of the predicted *A*. *sparsifolia* genes. Out of 24,639 NR hits and 16,417 PFAM homologies, 18,272 unigenes were matched into 3 main categories: biological process, cellular component, and molecular function (Fig. 4). Under the biological process category, the major GO terms were ‘cellular process’ (11,148, 61.0%), ‘metabolic process’ (10,597, 58.0%), ‘single-organism process’ (5,404, 29.6%), ‘biological regulation’ (3,668, 20.1%), ‘regulation of biological process’ (3414, 18.7%), ‘establishment of localization’ (3046, 16.7%) and ‘response to stimulus’ (2478, 13.6%). Within the cellular component category, a significant percentage of genes were clustered into ‘cell’ and ‘cell part’, both of which shared the same proportion (6471, 35.4%). In the molecular function category, most genes were assigned to ‘binding’ (11086, 60.7%) and ‘catalytic activity’ (9187, 50.3%) followed by transporter activity (1271, 7.0%), nucleic acid binding transcription factor activity (670, 3.7%) and structural molecule activity (560, 3.1%).

**Fig 5:**
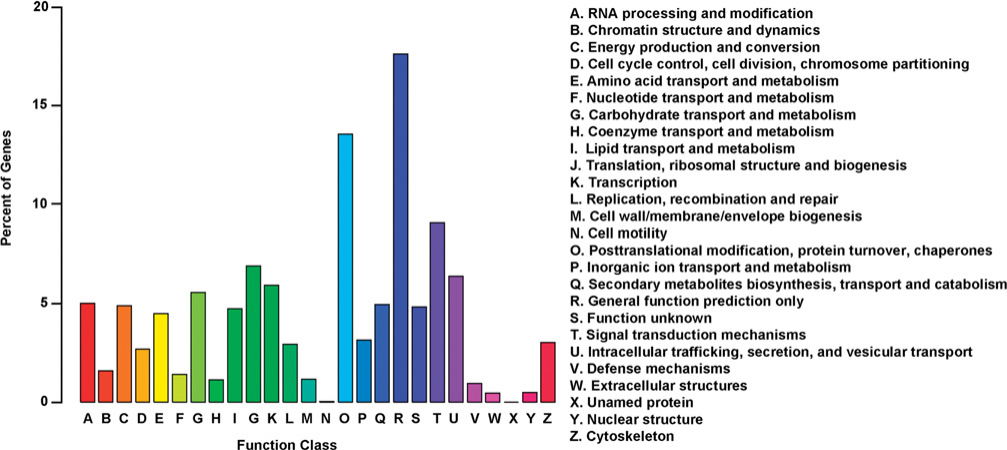
KOG function classification of the transcriptome of the *A*. *sparsifolia* primary roots. Out of 24,639 NR hits and 16,417 PFAM homologies, 9,313 sequences have a KOG classification among the 26 categories.

The proteins assigned to the same KOG category were assumed to have the common ancestor protein, or to be paralogs or orthologs. Using KOG, totally 9,313 unigenes were annotated and clustered into 26 categories (Fig. 5). Among them, the cluster for ‘General function prediction’ represented the largest group (1,641, 17.6%) followed by ‘Posttranslational modification, protein turnover, chaperones’ (1,263, 13.6%), ‘Signal transduction mechanism’ (843, 9.1%) and ‘Translation, ribosomal structure and biogenesis’ (641, 6.9%).

**Fig 6:**
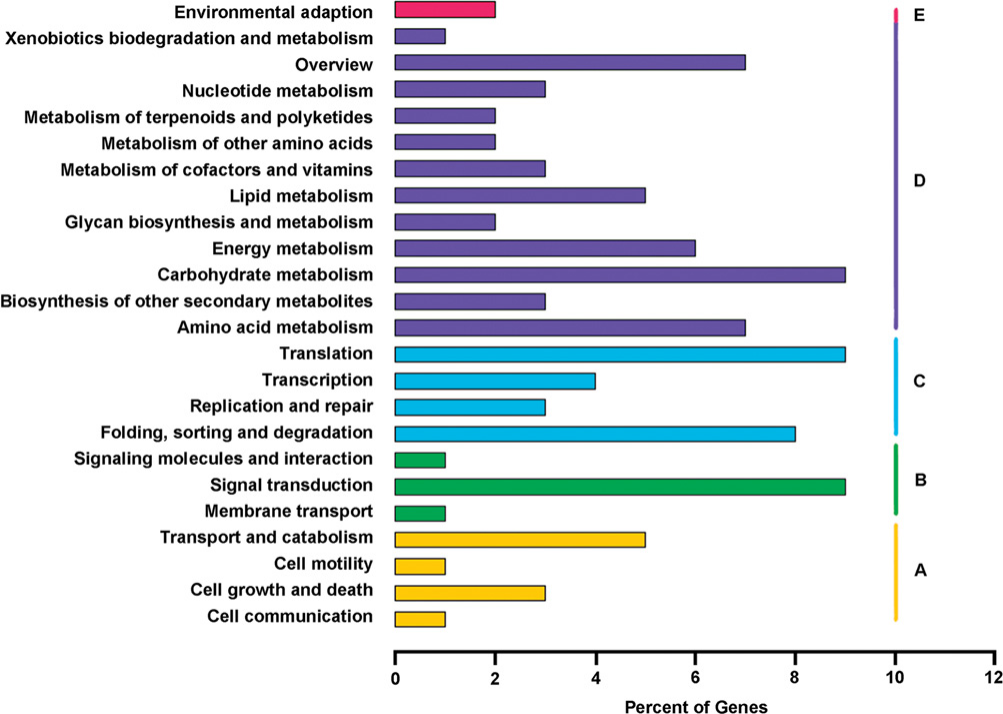
KEGG classification of assembled unigenes. A total of 6,452 unigenes were assigned to KEGG pathways of cellular processes (A), environmental information processing (B), genetic information processing (C), metabolism (D) and organism system (E).

KEGG pathway-based analyses help to systematically explore inner-cell metabolic pathways and biological functions of gene products. As shown in Fig. 6, a total of 6,452 unigenes were assigned to 24 subcategories in five KEGG biochemical pathways including cellular processes (708, 11.0%), environmental information processing (634, 9.8%), genetic information processing (1517, 23.5%), metabolism (3196, 49.5%) and organism system (158, 2.5%). Among them, the most dominant subcategory was identified as ‘Translation’ (595, 9.2%), followed by ‘Carbohydrate metabolism’ (588, 9.1%), ‘Signal transduction’ (565, 8.8%), ‘Folding, sorting and degradation’ (513, 8.0%), and ‘Amino acid metabolism’ (428, 6.6%).

### Global analysis of gene expression in *A*. *sparsifolia* primary roots under water stress and subsequent rehydration

To characterize the effect of water stress and subsequent rehydration on gene expression in the *A*. *sparsifolia* primary roots, the clean reads derived from P-0h, P-6h, P-24h and Rh-6h were individually mapped to the primary root transcriptome database containing 33,255 unigenes. For each group of the clean reads, over 80% were uniquely mapped to the reference database, thus identifying 26,452, 28,803, 29,012 and 29,746 unigenes expressed in P-0h, P-6h, P-24h and Rh-6h, respectively (Table 4).

**Table 4:** Statistics of clean reads mapped to the primary root transcriptome of *A*. *sparsifolia*.

Sample	Total clean reads	Unique match reads^a^	Reads-mapped genes^a^
P0_Root	38,763,230	31,701,920 (81.78%)	26,452 (76.4%)
P6_Root	67,511,150	57,981,060 (85.88%)	28,803 (81.8%)
P24_Root	49,259,804	42,724,090 (86.73%)	29,012 (83.7%)
Rh6_Root	54,774,906	46,490,800 (84.88%)	29,746 (87.6%)

a. The conservative degree of mismatch was no more than 2 bp.

The expression levels of the identified unigenes in P-0h, P-6h, P-24h and Rh-6h were further normalized with FPKM values [42], which takes the influence of both the sequencing depth and gene length on read count into account. As summarized in Table B in S1 File, among the four libraries of the expressed unigenes, the proportion of genes with FPKMs in interval of ‘>15’, the so-called high-level expressed genes, gradually increased in the order of P-0h, P-6h, P-24h and Rh-6h. In contrast, the percentage of genes with FPKMs in interval of ‘≦3.57’ representing the low-level expressed genes decreased gradually following the same order. Unambiguously, the unigene libraries of P-0h, P-6h, P-24h and Rh-6h together reflected the dynamics patterns of gene expression in *A*. *sparsifolia* primary roots in the course of 24h of water stress and subsequent 6h of rehydration, and offered an opportunity to identify the DEGs here below.

### Genes responding to water stress and subsequent rehydration in *A*. *sparsifolia* primary roots

**Fig 7:**
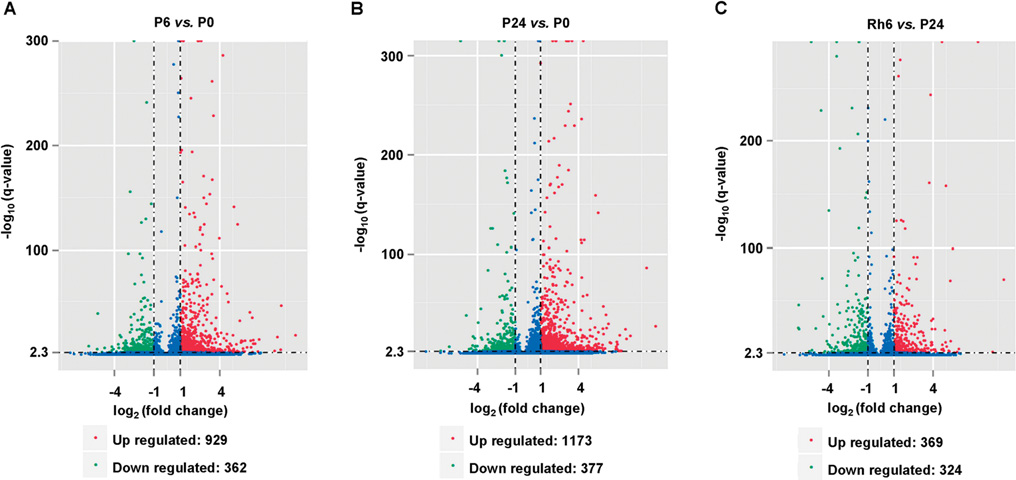
Gene expression profile changes in the *A*. *sparsifolia* primary roots during 24h of PEG treatment following 6h of rehydration. Comparisons between P6 and P0 (A), P24 and P0 (B), and Rh6 and P24 (C) were made on the basis of *q*-value<0.005 and |log2(foldchange)|>1. P0 represents the untreated primary roots. P6 and P24 represent the primary roots with 6h and 24h of PEG treatment, respectively. Rh6 represents the primary roots with 6h of rehydration preceded by 24h of PEG treatment. The identified up- and down-regulated genes are denoted as the red and green dots, and their numbers of are summarized.

The DEGs in response to water stress and the subsequent rehydration were explored by using DEGseq [44] with the criteria of significance [*q*-value<0.005, and |log2 (foldchange)|>1]. To this end, 929 up- and 362 down-regulated unigenes were detected from the P-6h library as compared to the P-0h library, and defined as the early water-stress inducible and repressed genes, respectively (Fig. 7A and Table C in S1 File). Between the libraries of P-24h and P-0h, 1,173 up- and 377 down-regulated unigenes were identified based on P-0h, and termed the late water-stress inducible and repressed genes, respectively (Fig. 7B and Table D in S1 File). In addition, 369 up- and 324 down-regulated genes were detected from the Rh-6h library as compared to the P-24h library, and named as the rehydration inducible and repressed genes, respectively (Fig. 7C and Table E in S1 File). Notably, there were more up-regulated unigenes than down-regulated ones in response to water stress, whereas almost equal amount of unigenes were up- and down-regulated under the rehydration condition, implying a positive role of the 220 g/L PEG solution on gene expression in the *A*. *sparsifolia* primary roots.

**Fig 8:**
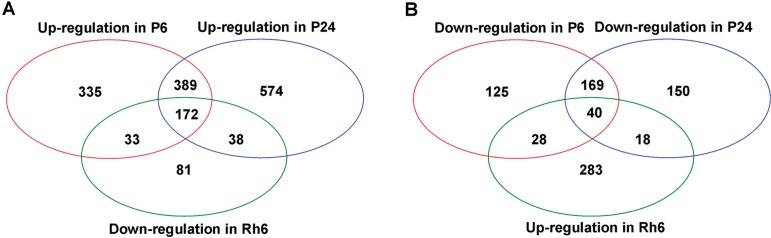
Venn diagrams showing the number of the common and specific DEGs in P-6h, P-24h and Rh-6h. The analysis were based either on genes up regulated in P-6h and P-24h (compared to P-0h) and down regulated in Rh-6h (compared to P-24h) (A), or on genes down regulated in P-6h and P-24h (compared to P-0h) and up regulated in Rh-6h (compared to P-24h) (B).

Based on the identified DEGs, the comparisons were first made to collect the genes exhibiting specific expression patterns in the course of water stress. As shown in Venn diagrams (Fig. 8A and 8B), there were 561 genes in common between the early and late water-stress inducible genes, and 209 genes in common between the early and late water-stress repressed genes (Tables F and G in S1 File). These overlapped genes were continuously induced or repressed following the perception of water stress, representing a general positive response to water stress in *A*. *sparsifolia* primary roots. On the other hand, 368 and 612 genes were identified to be exclusively in either dataset of the early water-stress inducible genes and late water-stress inducible genes (Tables H and I in S1 File), and 153 and 168 genes uniquely present in either of the early water-stress repressed genes and late water-stress repressed genes (Tables J and K in S1 File). This was not surprising that, along the general response, many genes were specifically regulated at different time points, showing temporal specific expression under the PEG-induced water stress. Interestingly, no common gene was identified between the early water-stress inducible genes and the late water-stress repressed genes, or between the early water-stress repressed genes and the late water-stress inducible genes, indicating that fewer genes exhibited opposite patterns of induction and repression at 6h and 24h of water stress.

Efforts were further done to capture the unigenes in particular showing alternative expression patterns under water stress conditions (6h and 24h of PEG treatment) as compared to 6h of rehydration. A total of 172 common genes were identified from the early and late water-stress inducible genes and the rehydration repressed genes, showing an ‘up-up-down’ expression pattern (Fig. 8A and Table L in S1 File). Conversely, among the early and late water-stress repressed genes and the rehydration inducible genes, there were 40 genes in common exhibiting a ‘down-down-up’ pattern (Fig. 8B and Table M in S1 File).

### Functional categorization of DEGs

**Fig 9:**
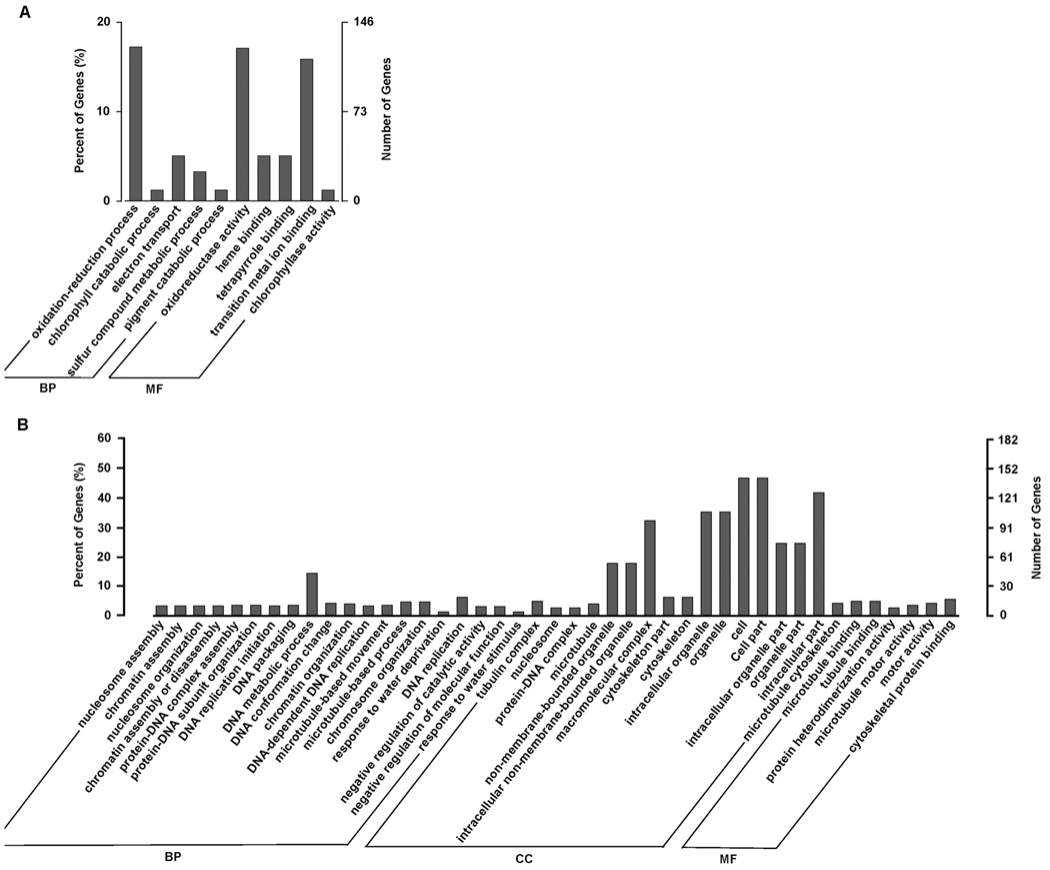
GO categories of biological process (BP), cellular component (CC) and molecular function (MF) for the early water-stress responding genes (P-6h *vs*. P-0h) in *A*. *sparsifolia* primary roots. (A) the early water-stress inducible genes; (B) the early water-stress repressed genes. The right y-axis shows the number of genes in a category, and the left y-axis indicates the percentage of a specific category of genes in that main category.

As an objective means of generalizing the biological functions represented by the significant change in gene expression, the GO category enrichment analysis by using GOseq [46] were preformed with the six datasets of DEGs including the early water-stress inducible and repressed genes, the late water-stress inducible and repressed genes, and the rehydration inducible and repressed genes. As shown in Fig. 9A, for the 730 early water-stress inducible genes with GO annotations, totally 10 GO terms were significantly enriched with a corrected p-value cut-off of 0.05 and clustered into the main categories of biological process and molecular function, but these genes did not reveal significantly enriched groups of genes belonging to any cellular component term. The ‘oxidation-reduction process’ comprising of 126 genes was the most dominant group in biological processes, and ‘oxidoreductase activity’ with 125 genes was dominant in molecular functions. In addition, genes associated with ‘chlorophyll catabolic process’, ‘electron transport’, ‘sulfur compound metabolic process’ and ‘pigment catabolic process’ were also enriched within the dataset.

**Fig 10:**
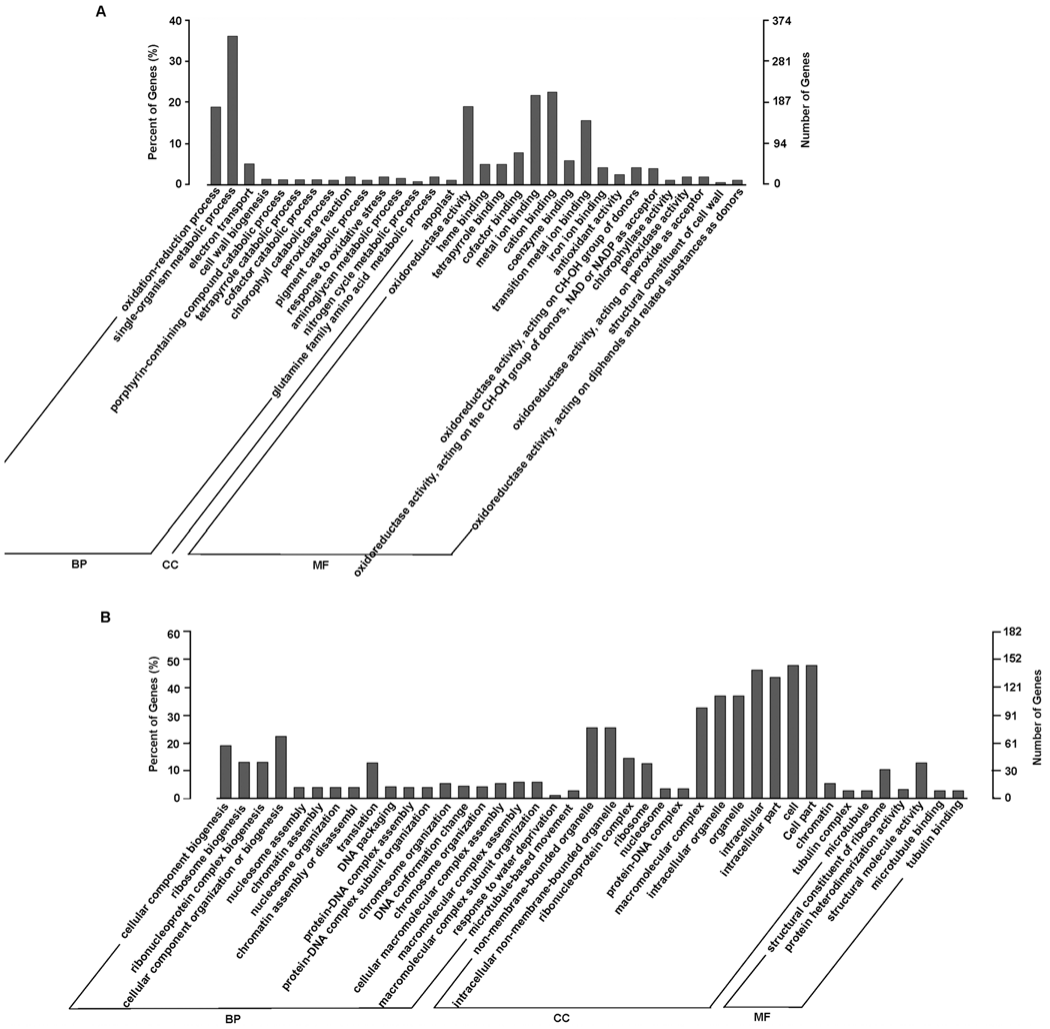
GO categories of biological process (BP), cellular component (CC) and molecular function (MF) for the late water-stress responding genes (P-24h *vs*. P-0h) in *A*. *sparsifolia* primary roots. (A) the late water-stress inducible genes; (B) the late water-stress repressed genes. The right y-axis shows the number of genes in a category, and the left y-axis indicates the percentage of a specific category of genes in that main category.

Likewise, for 936 late water-stress inducible genes with GO annotations, a total of 32 significantly enriched GO terms were also predominantly clustered into biological process and molecular function. The ‘apoplast’ was the unique enriched subcategory under cellular component (Fig. 10A). Among the biological processes, the ‘oxidation-reduction process’ with 177 genes remained the dominant group, and a significant number of genes associated with oxidoreductase activity were enriched in terms of molecular function. The additional enriched GO terms under biological process included ‘cell wall biogenesis’, ‘single-organism metabolic process’, ‘electron transport’, ‘peroxidase reaction’, ‘response to oxidative stress’, and ‘glutamine family amino acid metabolic process’.

In contrast to the relative narrow distribution of the early and late water-stress inducible genes in GO category enrichment, the early and late water-stress repressed genes were individually enriched into 43 and 41 GO terms clustered in all of the three main categories (Figs. 9B and 10B), though each of the two datasets had only 303 GO-annotated genes, much fewer than the early and the late water-stress inducible genes with GO annotations. In particular, a significant number of genes were enriched into a broad range of cellular component terms, with ‘cell’ and ‘cell part’ as the most represented ones. These implied that water stress might repress a broad spectrum of the transcriptome in *A*. *sparsifolia* primary roots.

**Fig 11:**
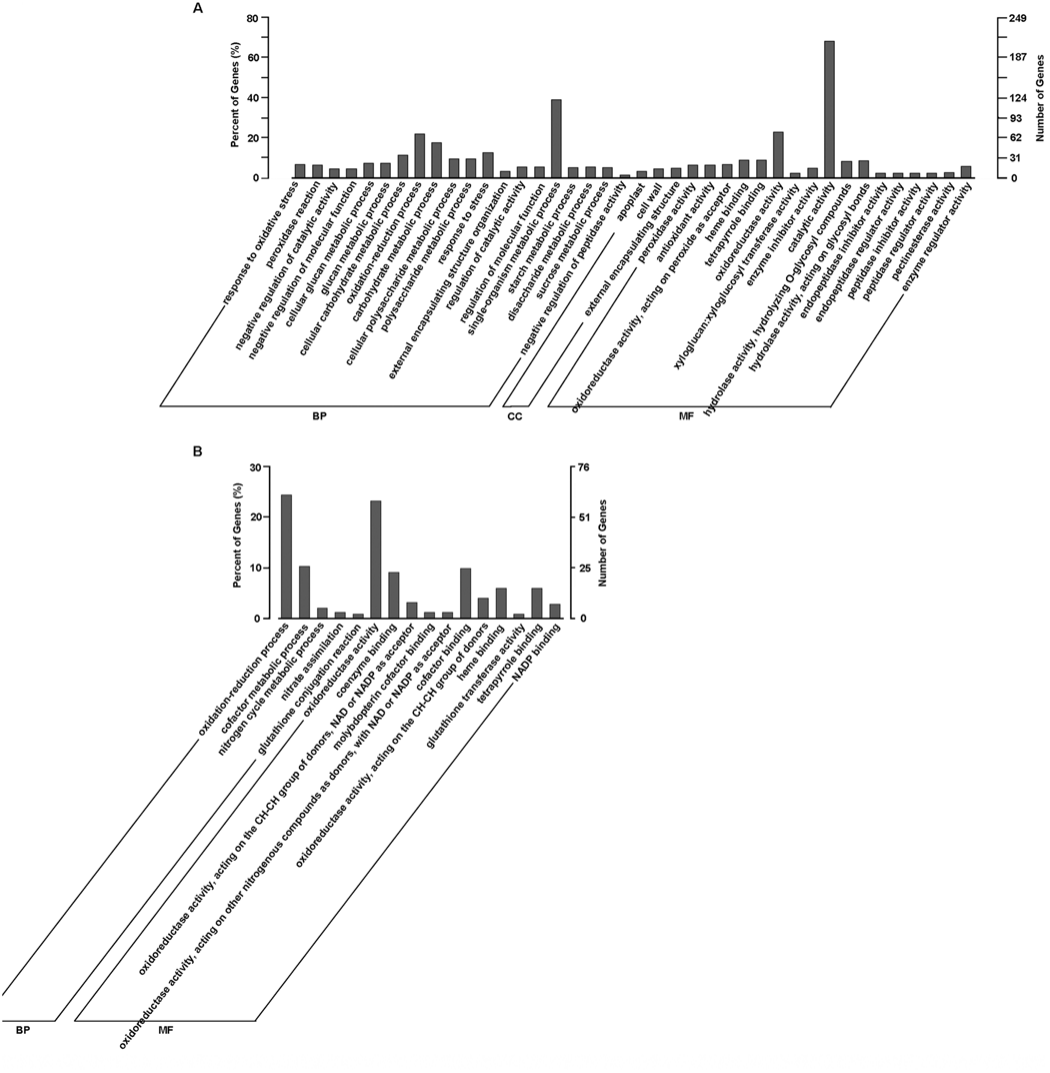
GO categories of biological process (BP), cellular component (CC) and molecular function (MF) for the rehydration responding genes (Rh-6h *vs*. P-24h) in *A*. *sparsifolia* primary roots. (A) the rehydration inducible genes; (B) the rehydration repressed genes. The right y-axis shows the number of genes in a category, and the left y-axis indicates the percentage of a specific category of genes in that main category.

The rehydration inducible and repressed genes, however, showed a completely reverse pattern in GO category enrichment when compared to the water-stress inducible and repressed genes. As shown in Fig. 11A, for 311 rehydration inducible genes with GO annotations, there were 40 significantly enriched GO terms in all three main categories. The ‘single-organism metabolic process’ with 121 genes were dominant in biological process, a high percentage (68.17%) of genes were categorized into ‘catalytic activity’ under molecular function, and ‘external encapsulating structure’ with 15 genes, ‘cell wall’ with 14 genes and ‘apoplast’ with 10 genes were of the three enriched cellular_component terms. For 254 rehydration repressed genes with GO annotations, however, only 16 GO terms were significantly enriched and clustered into biological process and molecular function (Fig. 11B). Among them, ‘oxidation-reduction process’ with 62 genes in biological process and ‘oxidoreductase activity’ with 59 genes in molecular function were dominant.

To ascertain the biological pathways that are active in *A*. *sparsifolia* primary roots during water stress and subsequent rehydration, the identified DGEs were further mapped to reference canonical pathways in KEGG database with KOBAS [47], and compared these with the whole transcriptome background, with a view of searching the significantly enriched metabolic or signal transduction pathways. Numbers of KEGG pathways were individually determined on the early water-stress inducible and repressed genes, the late water-stress inducible and repressed genes, and the rehydration inducible and repressed genes, and the significant ones were further filtered with a corrected p-value cut-off of 0.05 (Table N in S1 File). As a result, the ‘Glutathione metabolism pathway’ was identified as the most significant KEGG pathway from the early water-stress inducible genes, the late water-stress inducible genes as well as the rehydration repressed genes. Notably, this was the only significantly enriched pathway in both the early water-stress inducible genes and the rehydration repressed genes. Moreover, within the ‘Glutathione metabolism pathway’, there were five associated genes, including glutathione *S*-transferase (GST), glucose-6-phosphate dehydrogenase (G6PDH), 6-phosphogluconate dehydrogenase (6PGD), L-ascorbate peroxidase and spermidine synthase, which were represented by eight unigenes with the ‘up-up-down’ expression pattern, suggesting their dynamic expression pattern in the course of the water stress and the following rehydration. Besides the ‘Glutathione metabolism pathway’, four additional pathway, such as ‘Metabolism of xenobiotics by cytochrome P450’, ‘Drug metabolism-cytochrome P450’, ‘Alanine, aspartate and glutamate metabolism’ and ‘Microbial metabolism in diverse environments’, were also significantly enriched from the late water-stress inducible genes, suggesting the complexity of the biological pathways changes under long-term water stress.

### Validation of DEGs with qRT-PCR

**Fig 12:**
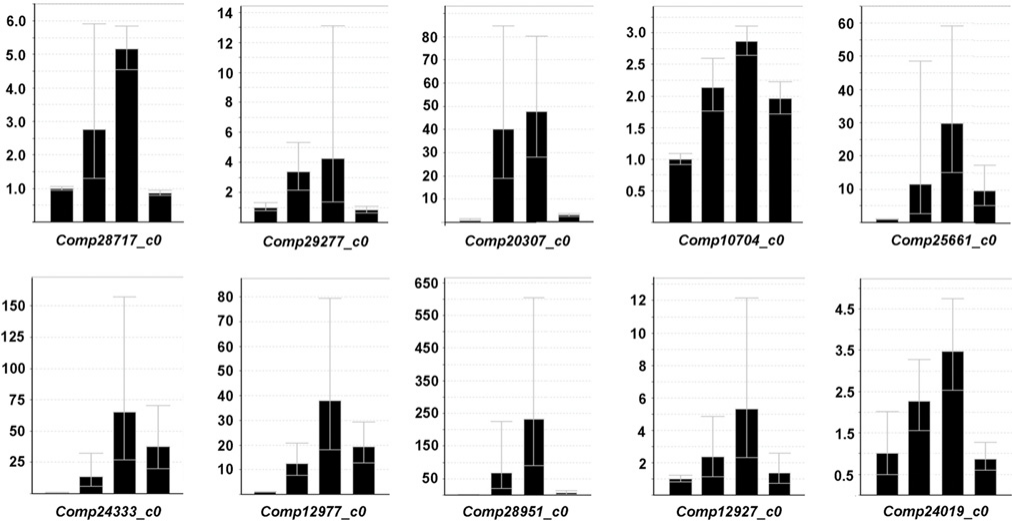
Validation of the DEGs by RT-qPCR. The relative expression levels of ten unigenes, which exhibited the ‘up-up-down’ expression pattern in the course of 24h of water stress following 6h of rehydration, were calculated with the 2^–ΔΔCT^ method. The *actin* gene was used as an internal control. For each tested unigene, the columns from left to right represented its relative expressive levels in P-0h, P-6h, P-24h and Rh-6h, respectively. Error bars indicated the standard deviation of the mean expression values.

To validate the differential expression data revealed by RNA-Seq, the RT-qPCR assay was performed on 10 unigenes which demonstrate the ‘up-up-down’ expression pattern in the course of 24h of water stress following 6h of rehydration (Table N in S1 File). The selected unigenes, with the exception of the ones encoding uncharacterized or hypothetical proteins, were annotated with sulfate transporter, inorganic phosphate transporter 1–4, 6PGD, G6PDH, GST, nitrate reductase 1 and auxin induced protein, respectively. As shown in Fig. 12, all tested transcripts showed similar expression patterns to those from high-throughput RNA sequencing, suggesting a strong correlation. Hence, the RNA-Seq data were considerably reliable for identification of DEGs in this study.

## Discussion

*A*. *sparsifolia* is an ecologically important woody plant species occurring across the desert ecosystems of Mid-Asia, and exhibits substantial tolerance under extreme drought stress conditions [31,32]. However, as a non-model plant species, the genomic resource of *A*. *sparsifolia* is rather scarce, only one expressed sequence tag (EST) and one protein have been deposited in Genbank prior to Jan., 2015. Understanding of the molecular mechanism underlying the drought tolerance of *A*. *sparsifolia* is greatly impeded and delayed. In the current study, we first report the transcriptomic analyses conducted on primary roots of *A*. *sparsifolia*, and identify numbers of DEGs responding to the early water stress, the late water stress as well as the immediate rehydration after water stress, thereby offering an opportunity to view the sequence resources of *A*. *sparsifolia*, and in particular the dynamic profile of gene expression in its primary roots under water stress.

On the basis of GO category enrichment analysis of the DEGs, we observed that the ‘oxidation-reduction process’ present as a dominant term in both the early water-stress inducible genes and the late water-stress inducible genes. Given the essential role of the oxidoreductase system in stress response [49,50], it was not surprising that the unigenes associated with the oxidoreductase system were significantly induced upon water deficit in primary roots of *A*. *sparsifolia*. Interestingly, the ‘oxidation-reduction process’ remained dominant in the group of the rehydration repressed genes, suggesting that the active oxidoreductase system under water stress could be down regulated once under the well watered conditions. Nevertheless, the data together conclusively demonstrated the prime importance of the oxidoreductase system in primary roots of *A*. *sparsifolia* in response to water stress. In addition, consistent with the previous finding that sulfur is one of crucial factors determining abiotic stress tolerance in plant [51,52], in this study, 24 unigenes associated with ‘sulfur compound metabolic process’ were enriched from the early water-stress inducible genes, suggesting their critical role in the early response to water stress in *A*. *sparsifolia* primary roots. GO analysis also showed a strong enrichment of 12 unigenes associated with ‘cell wall biogenesis’ in the late water-stress inducible genes. This implied the occurrence of cell wall remodeling in *A*. *sparsifolia* primary roots upon water stress, providing further evidence that maintenance of cell wall integrity is essential for stress response [53,54].

A particular interest was also given to the DEGs whose expression alternatively switched between induction and repression depending on water deficit or well watered conditions. We identified 172 unigenes, the expression of which was significantly up regulated at the early and late stages of water stress but repressed under the rehydration. This specific ‘up-up-down’ expression pattern strongly suggested their implication on positive regulation of the water stress response. Within this dataset, many unigenes were homologs of those have been well documented to function in biotic/abiotic stress tolerance in plants [7,55–59], such as heat shock protein, late embryogenesis abundant protein, cytochrome P450, dehydrin, transcription factors (NAC family members, bZIP, ethylene-responsive factor and zinc finger protein), and hormone-related protein (9-cis-epoxycarotenoid dioxygenase and auxin-induced protein). In contrast, 40 unigenes were characterized to be repressed across the early and late stages of water stress but up regulated under the rehydration. *as per* NR and GO annotation, the genes within this dataset included proline dehydrogenase, serine/threonine protein kinase, MYB transcription factor, xyloglucan:xyloglucosyl transferase and pectinesterase. Regarding their unique expression pattern of ‘down-down-up’, these unigenes should represent the negative regulators of the water stress response in *A*. *sparsifolia* primary roots. In fact, proline dehydrogenase, as the first and rate-limiting enzyme of proline degradation, has been known to be repressed under water deficit but induced under hypoosmolarity [60,61]. Additionally, the MYB transcription factors with necessary roles in negative regulation of abiotic stress response were also characterized [62–64].

As far as is known that glutathione is a major substrate in antioxidative defense mechanisms by quenching reactive oxygen species, eliminating peroxides and scavenging free radicals [65–67]. The enhancement of glutathione metabolism in response to water stress has been reported in many plant species, such as Arabidopsis [68], chickpea [22], *Gossypium herbaceum* [24] and poplar [29]. In this study, KEGG pathway enrichment analysis of DEGs revealed that the ‘Glutathione metabolism pathway’ was of the most significant pathway associated with not just the early and the late water-stress inducible genes but also the rehydration repressed genes. This clearly disclosed that regulation of this pathway in *A*. *sparsifolia* primary roots seriously depended on the water supply conditions, indicating significance of the glutathione metabolism in *A*. *sparsifolia* primary roots against water stress.

It was worthy to emphasize that GST, G6PDH, 6PGD, L-ascorbate peroxidase and spermidine synthase, the five genes associated with the ‘Glutathione metabolism pathway’ showed the expression pattern of ‘up-up-down’. As reported previously, GST is one of the most important catalytic enzymes that utilize glutathione to function in the stress tolerance mechanisms of plant [69], and G6PDH and 6PGD, as two kinds of dehydrogenase, are responsible for reduction of NADP+ to NADPH [70], thereby playing critical roles in glutathione maintenance under stress conditions [71]. In addition, spermidine synthase is known to be a key enzyme in biosynthesis of spermidine, a kind of polyamines that have been implicated in stress tolerance by ROI scavenging and membrane protection [72], but recent progress showed that the polyamine spermidine can also conjugate with glutathione to form the antioxidant metabolite trypanothione [N(1),N(8)-bis(glutathionyl)spermidine] against chemical and oxidant stress [73,74]. Likewise, L-ascorbate peroxidase does not directly utilize glutathione as substrate but has a central role to maintain the ascorbate/glutathione cycle, wherein ascorbate is coupled with glutathione, and thus more effectively regulates the cellular H_2_O_2_ metabolism [75]. Along with these previous observations, the data in this study indicated that the five aforementioned genes might act as gene switches functioning synergistically to regulate the glutathione metabolism in *A*. *sparsifolia* primary roots depending on water conditions.

## Conclusions

This report represents the first application of Illumina sequencing technology for transcriptome studies in primary roots of *A*. *sparsifolia*, a typical desert plant with extreme drought tolerance. A total of 33,255 unigenes were generated with a depth of 21.04 gigabase pairs, and 24,639 unigenes were functionally annotated with NR database. Based on this transcriptome database, we further explored the gene expression profiles in *A*. *sparsifolia* primary roots during 24h of water stress following 6h of rehydration, and defined numbers of unigenes specifically responding to water stress. The subsequent functional enrichment disclosed a large number of water-stress related genes associated with oxidoreductase system, and in particular highlighted the impact of glutathione metabolism on water stress, thus providing potential targets for candidate gene selection in improving the water stress tolerance of plants.

## Supporting Information

S1 File The file includes Table A, Table B, Table C, Table D, Table E, Table F, Table G, Table H, Table I, Table J, Table K, Table L, Table M and Table N. Table A in S1 File. Primers used in the RT-qPCR assay. Table B in S1 File. Statistics of unigenes in different expression-level interval. Table C in S1 File. Differentially expressed genes between P-6h and the untreated primary roots P-0h (early water-stress inducible and repressed genes). The significance of gene expression difference between samples was identified by using *q*-value<0.005 and the absolute value of log2 (foldchange)>1 as the threshold. Table D in S1 File. Differentially expressed genes between P-24h and the untreated primary roots P-0h (late water-stress inducible and repressed genes). The significance of gene expression difference between samples was identified by using *q*-value<0.005 and the absolute value of log2 (foldchange)>1 as the threshold. Table E in S1 File. Differentially expressed genes between Rh-6h and P-24h (rehydration inducible and repressed genes). The significance of gene expression difference between samples was identified by using *q*-value<0.005 and the absolute value of log2 (foldchange)>1 as the threshold. Table F in S1 File. Common genes between the early and late water-stress inducible genes. Table G in S1 File. Common genes between the early and late water-stress repressed genes. Table H in S1 File. Genes exclusively expressed as the early inducible genes. Table I in S1 File. Genes exclusively expressed as the late water-stress inducible genes. Table J in S1 File. Genes exclusively expressed as the early water-stress repressed genes. Table K in S1 File. Genes exclusively expressed as the late water-stress repressed genes. Table L in S1 File. Genes showed ‘up-up-down’ expression pattern in the course of 24h of PEG-treatment following 6h of rehydration. Table M in S1 File. Genes showed ‘down-down-up’ expression pattern in the course of 24h of PEG-treatment following 6h of rehydration. Table N in S1 File. The significant KEGG pathways enriched from the early and late water-stress inducible genes (P-6h *vs*. P-0h) the early and late water-stress repressed genes (P-24h *vs*. P-0h), the rehydration inducible and repressed genes (Rh-6h *vs*. P-24h). The corrected p-value cut-off of 0.05 was set as the threshold, and only the enriched pathways with p-value less than 0.05 were shown.
(ZIP)
