# Supplementary material for: Transcriptomic Analysis of the Primary Roots of Alhagi sparsifolia in Response to Water Stress
Source: PLoS One. 2015 Mar 30;10(3):e0120791. doi: 10.1371/journal.pone.0120791 (PMC4379016; doi:10.1371/journal.pone.0120791)
Supplement: S1 File — Table A in S1 File. Primers used in the RT-qPCR assay. Table B in S1 File. Statistics of unigenes in different expression-level interval. Table C in S1 File. Differentially expressed genes between P-6h and the untreated primary roots P-0h (early water-stress inducible and repressed genes). The significance of gene expression difference between samples was identified by using q-value<0.005 and the absolute value of log2 (foldchange)>1 as the threshold. Table D in S1 File. Differentially expressed genes between P-24h and the untreated primary roots P-0h (late water-stress inducible and repressed genes). The significance of gene expression difference between samples was identified by using q-value<0.005 and the absolute value of log2 (foldchange)>1 as the threshold. Table E in S1 File. Differentially expressed genes between Rh-6h and P-24h (rehydration inducible and repressed genes). The significance of gene expression difference between samples was identified by using q-value<0.005 and the absolute value of log2 (foldchange)>1 as the threshold. Table F in S1 File. Common genes between the early and late water-stress inducible genes. Table G in S1 File. Common genes between the early and late water-stress repressed genes. Table H in S1 File. Genes exclusively expressed as the early inducible genes. Table I in S1 File. Genes exclusively expressed as the late water-stress inducible genes. Table J in S1 File. Genes exclusively expressed as the early water-stress repressed genes. Table K in S1 File. Genes exclusively expressed as the late water-stress repressed genes. Table L in S1 File. Genes showed ‘up-up-down’ expression pattern in the course of 24h of PEG-treatment following 6h of rehydration. Table M in S1 File. Genes showed ‘down-down-up’ expression pattern in the course of 24h of PEG-treatment following 6h of rehydration. Table N in S1 File. The significant KEGG pathways enriched from the early and late water-stress inducible genes (P-6h vs. P-0h) the early and late [file pone.0120791.s001.zip › Table A in S1 File.doc]

**Table A in S1 File.** Primers used in the RT-qPCR assay.

| **Unigene ID** | **Gene name** | **Fragment(bp)** | **Direction** | **Primer sequence (5'-3')** |
| --- | --- | --- | --- | --- |
| comp28717_c0 | 6PGD | 119 | Forward | GGGTTGTGGGGTTGGCTATT |
|  |  |  | Reverse | CCCTCTGAGCCTGAACAAGG |
| comp29277_c0 | G6PDH | 154 | Forward | GGAGTCTCAAGGTGAAGCCT |
|  |  |  | Reverse | GGTGAAGTGCTTAGGGAGACA |
| comp10704_c0 | GST | 134 | Forward | TCTTGGAGAACGCTCTTGGT |
|  |  |  | Reverse | GATGTCATGCTTGAACGCCTC |
| comp20307_c0 | sulfate transporter | 168 | Forward | TGCTTGGGTATATTCAGGCTGG |
|  |  |  | Reverse | GTGCTTCACCACTGCTACAAC |
| comp25661_c0 | unknown | 183 | Forword | ACTAGTAGCAGCTCGCCATC |
|  |  |  | Reverse | GAGGGCATGTTAGGAAGTGGA |
| comp24333_c0 | unknown protein | 171 | Forward | GCTGCCCAAGGTTCCTGAAT |
|  |  |  | Reverse | GGGACCTTAGACAATTCTGGCT |
| comp12977_c0 | uncharacterized protein | 188 | Forward | ATGGACACTAGCCTTGCAGC |
|  |  |  | Reverse | TGTGGGACACTTGGAATGGT |
| comp28951_c0 | nitrate reductase 1 | 101 | Forward | GCTCAAGCGCTGTGGAATTT |
|  |  |  | Reverse | GCCTGAGACAGCAAGGTACA |
| comp24019_c0 | Auxin induced protein | 137 | Forward | TGGCTCTACCCCTCAGAGAT |
|  |  |  | Reverse | CATCGACAGAACACGGAAGC |
| comp26182_c0 | actin | 169 | Forward | GCGGGAAATTGTTCGTGACA |
|  |  |  | Reverse | AAGAACTTCTGGGCAACGGA |
