# Supplementary material for: Transcriptomic Analysis of the Primary Roots of Alhagi sparsifolia in Response to Water Stress
Source: PLoS One. 2015 Mar 30;10(3):e0120791. doi: 10.1371/journal.pone.0120791 (PMC4379016; doi:10.1371/journal.pone.0120791)
Supplement: S1 File — Table A in S1 File. Primers used in the RT-qPCR assay. Table B in S1 File. Statistics of unigenes in different expression-level interval. Table C in S1 File. Differentially expressed genes between P-6h and the untreated primary roots P-0h (early water-stress inducible and repressed genes). The significance of gene expression difference between samples was identified by using q-value<0.005 and the absolute value of log2 (foldchange)>1 as the threshold. Table D in S1 File. Differentially expressed genes between P-24h and the untreated primary roots P-0h (late water-stress inducible and repressed genes). The significance of gene expression difference between samples was identified by using q-value<0.005 and the absolute value of log2 (foldchange)>1 as the threshold. Table E in S1 File. Differentially expressed genes between Rh-6h and P-24h (rehydration inducible and repressed genes). The significance of gene expression difference between samples was identified by using q-value<0.005 and the absolute value of log2 (foldchange)>1 as the threshold. Table F in S1 File. Common genes between the early and late water-stress inducible genes. Table G in S1 File. Common genes between the early and late water-stress repressed genes. Table H in S1 File. Genes exclusively expressed as the early inducible genes. Table I in S1 File. Genes exclusively expressed as the late water-stress inducible genes. Table J in S1 File. Genes exclusively expressed as the early water-stress repressed genes. Table K in S1 File. Genes exclusively expressed as the late water-stress repressed genes. Table L in S1 File. Genes showed ‘up-up-down’ expression pattern in the course of 24h of PEG-treatment following 6h of rehydration. Table M in S1 File. Genes showed ‘down-down-up’ expression pattern in the course of 24h of PEG-treatment following 6h of rehydration. Table N in S1 File. The significant KEGG pathways enriched from the early and late water-stress inducible genes (P-6h vs. P-0h) the early and late [file pone.0120791.s001.zip › Table B in S1 File.doc]

**Table B in S1 File.** Statistics of unigenes in different expression-level interval.

| **FPKM Interval** | **P0_Root** | **P6_Root** | **P24_Root** | **Rh6_Root** |
| --- | --- | --- | --- | --- |
| 0-0.1 | 6,892 (20.72%) | 4,591 (13.81%) | 4,302 (12.94%) | 3,527 (10.61%) |
| 0.1-0.3 | 949 (2.85%) | 1,445 (4.35%) | 1,108 (3.33%) | 610 (1.83%) |
| 0.3-3.57 | 12,458 (37.46%) | 14,220 (42.76%) | 13,660 (41.08%) | 13,480 (40.54%) |
| 3.57-15 | 7,480 (22.49%) | 6,886 (20.71%) | 7,210 (21.68%) | 7,901 (23.76%) |
| 15-60 | 3,840 (11.55%) | 3,968 (11.93%) | 4,631 (13.93%) | 5,286 (15.90%) |
| >60 | 1,636 (4.92%) | 2,145 (6.45%) | 2,344 (7.05%) | 2,451 (7.37%) |
